# *Lactobacillus reuteri* in digestive system diseases: focus on clinical trials and mechanisms

**DOI:** 10.3389/fcimb.2023.1254198

**Published:** 2023-08-18

**Authors:** Yijing Peng, Yizhe Ma, Zichen Luo, Yifan Jiang, Zhimin Xu, Renqiang Yu

**Affiliations:** ^1^ Department of Neonatology, Women’s Hospital of Jiangnan University, Wuxi Maternity and Child Health Care Hospital, Wuxi, China; ^2^ Wuxi Children’s Hospital, Children’s Hospital of Jiangnan University, Wuxi, China; ^3^ Department of Pediatric, Jiangyin People’s Hospital of Nantong University, Wuxi, China; ^4^ School of Medicine, Nantong University, Nantong, China; ^5^ College of Resources and Environment, Innovative Institute for Plant Health, Zhongkai University of Agriculture and Engineering, Guangzhou, China; ^6^ Research Institute for Reproductive Health and Genetic Diseases, Women’s Hospital of Jiangnan University, Wuxi Maternity and Child Health Care Hospital, Wuxi, China

**Keywords:** *Lactobacillus reuteri*, gut microbiota, infantile colic, diarrhea, constipation, functional abdominal pain, inflammatory bowel disease, colorectal cancer

## Abstract

**Objectives:**

Digestive system diseases have evolved into a growing global burden without sufficient therapeutic measures. *Lactobacillus reuteri* (*L. reuteri*) is considered as a new potential economical therapy for its probiotic effects in the gastrointestinal system. We have provided an overview of the researches supporting various *L. reuteri* strains’ application in treating common digestive system diseases, including infantile colic, diarrhea, constipation, functional abdominal pain, *Helicobacter pylori* infection, inflammatory bowel disease, diverticulitis, colorectal cancer and liver diseases.

**Methods:**

The summarized literature in this review was derived from databases including PubMed, Web of Science, and Google Scholar.

**Results:**

The therapeutic effects of *L. reuteri* in digestive system diseases may depend on various direct and indirect mechanisms, including metabolite production as well as modulation of the intestinal microbiome, preservation of the gut barrier function, and regulation of the host immune system. These actions are largely strain-specific and depend on the activation or inhibition of various certain signal pathways. It is well evidenced that *L. reuteri* can be effective both as a prophylactic measure and as a preferred therapy for infantile colic, and it can also be recommended as an adjuvant strategy to diarrhea, constipation, *Helicobacter pylori* infection in therapeutic settings. While preclinical studies have shown the probiotic potential of *L. reuteri* in the management of functional abdominal pain, inflammatory bowel disease, diverticulitis, colorectal cancer and liver diseases, its application in these disease settings still needs further study.

**Conclusion:**

This review focuses on the probiotic effects of *L. reuteri* on gut homeostasis via certain signaling pathways, and emphasizes the importance of these probiotics as a prospective treatment against several digestive system diseases.

## 1 Introduction

Digestive system diseases arise rapidly through the interaction of genetic and environmental characteristics and are considered to cause considerable healthcare burdens and costs (Peery et al., 2022; Ricciardiello, 2022). Based on the latest data, digestive system diseases are responsible for millions of healthcare encounters and thousands and thousands of deaths that cost billions of dollars in the United States every year (Peery et al., 2022). Of these digestive system diseases, inflammatory bowel disease (IBD) is estimated to affect 790 per 100,000 people in 2025 (Kaplan, 2015). According to the most recent worldwide estimates, there were 930,000 deaths of colorectal cancer (CRC) in 2020 and over 1.9 million new cases (Morgan et al., 2023). The similar phenomenon of increasing incidence is also seen in infantile colic (IC), functional abdominal pain (FAP), and other gastrointestinal (GI) diseases (Thapar et al., 2020; Peery et al., 2022; Indrio et al., 2023). Given this situation, there is an urgent need to deploy personalized treatments for these diseases.

Nowadays public interest of probiotics applications in therapeutic settings is growing. One of the most widely used probiotics, *Lactobacillus*, can be detected in the GI tract of animals in varying concentrations depending on the species, age of the host, or placement inside the gut (Zhao et al., 2023). The *Lactobacillus* is a large heterogeneous set of facultative anaerobic bacteria that are nonsporulating, and Gram-positive comprising *Lactobacillus (L.) acidophilus*, *L. reuteri, L. casei*, *L. bulgaricus*, *L. rhamnosus*, and so on (Mu et al., 2018). Among these, *L. reuteri* is an extensively investigated probiotic that was first isolated in 1962, resides in tissues of numerous mammals, and provides multiple health benefits for the host including producing antimicrobial molecules and regulating the host immune system (Yu et al., 2023b). *L. reuteri* was discovered in various body sites, which included breast milk, skin, urinary tract, and GI tract (Yu et al., 2023b). Numerous *L. reuteri* strains were found to play unique roles in different diseases covering hypercholesterolemia, skin infection, allergic asthma, periodontitis, and autism spectrum disorders as essential probiotics, especially in GI diseases (Sgritta et al., 2019; Kubota et al., 2020; Dargenio et al., 2021). Mounting evidence indicates that the symbiotic gut microbiota and host immune system work together to preserve gut homeostasis (Saeed et al., 2022). Growing data has revealed that the etiology of disorders of the digestive system is strongly influenced by disrupted gut microbes (Quaglio et al., 2022). Various *L. reuteri* strains have been investigated in digestive system diseases like IC, diarrhea, constipation, FAP, IBD, CRC, as well as liver diseases, and the findings are generally promising (Dias et al., 2021; Saviano et al., 2021; Werlinger et al., 2022; Happy Tummy et al., 2023).

But there are still doubts about clinical application of *L. reuteri*, so it is important to understand the underlying mechanism of *L. reuteri* in gut health. The effects of *L. reuteri* on GI diseases may include preservation of gut barrier function, suppression of excessive immune responses as well as oxidative stress, modification of the composition of the intestinal flora, and production of metabolites. In this review, we discussed the application of *L. reuteri* in digestive system diseases and outlined the potential mechanisms.

## 2 Clinical application of *L. reuteri* in digestive system diseases

### 2.1 Infantile colic

IC is typically characterized by daily full-force crying for a minimum of 3 hrs, on a minimum of 3 days per week, for at least 3 weeks (Gordon et al., 2018). It is reported about one in four infants younger than three months develop colic (Indrio et al., 2023). Although IC often resolves by three months of age, it remains to be stressful for parents and may even cause maternal postpartum depressive symptoms (Mercuri et al., 2023). Considerable research indicates that the intestinal microbiome’s composition is related to infant colic symptoms, even though the pathophysiology of this digestive condition is still inadequately comprehended (Korpela et al., 2020). Firstly, it has been hypothesized that the developing GI microbiome and the occurrence of excessive crying in infants between the ages of two weeks and three months-the normal time frame during which the gut is typically colonized by bacteria are related (Zeevenhooven et al., 2018). Additionally, the gut microbiota interacts with the gut-brain axis through multiple signaling pathways, including neural, endocrine, immune, and humoral signaling pathways (Deng et al., 2021). Given this, it may influence central and gut neural functions, such as pain perception in infants via the gut-brain axis, which may be associated with excessive crying (Socala et al., 2021). In the past decades, numerous studies illustrated that the GI microbial profiles of infants with colic differ from those without colic in microbial diversity, stability, and community composition (de Weerth et al., 2013; Savino et al., 2017). Moreover, the results showed colicky infants were less frequently colonized by *Lactobacillus*, *Bifidobacterium* and more frequently enriched with the gas-forming *Coliforms* (mostly *Escherichia*, *Klebsiella*) or other species (Savino et al., 2009; de Weerth et al., 2013; Savino et al., 2017; Sommermeyer et al., 2022; Kozhakhmetov et al., 2023).

We concluded that *L. reuteri* may be useful in the improvement of breastfeeding babies with IC based on six randomized controlled trials (RCTs), shown in **Table 1**. Accordingly, administering 1×10^8^ colony-forming units (CFU) of *L. reuteri* DSM 17938 per day for 21 to 30 days can significantly decrease daily crying and fussing times, and reduce the duration of crying episodes in colicky breast-fed infants, consistent with remission of maternal depression (Savino et al., 2010; Szajewska et al., 2013; Chau et al., 2015; Mi et al., 2015; Savino et al., 2018a; Savino et al., 2018b). Similar results were reported when analyzing the probiotic potential of *L. reuteri* ATCC 55730 in colicky infants, the superiority was still maintained compared with simethicone intervention (Savino et al., 2007). However, whether *L. reuteri* is useful in the treatment of formula-fed colicky infants is still controversial. Data shown in the other two RCT studies demonstrated that *L. reuteri* did not help manage the symptoms of colicky infants (Sung et al., 2014; Turco et al., 2021). One study showed that a partially hydrolyzed formula added with maltodextrins and *L. reuteri* DSM 17938 showed no preference for the standard formula on colicky symptoms, but the unsatisfactory results may attribute to the addition of maltodextrins in the probiotics group (Turco et al., 2021). In another study included breastfed and formula-fed colicky newborns, researchers found that *L. reuteri* DSM 17938 had no beneficial impact on crying or fusing time, instead, more fussing occurred in formula-fed infants following probiotic treatment (Sung et al., 2014). The conflict results may be explained by the differences in gut microbiota, whose composition in early infancy seemed to be influenced by various factors, including the delivery mode, infant feeding pattern, gestational age, and the history of antibiotic use (Kapourchali and Cresci, 2020). Formula-fed babies exhibited an increased gut bacterial community richness relative to breast-fed babies, with a greater abundance of *Clostridium difficile* (Azad et al., 2013). Following the administration of *L. reuteri* DSM 17938, breast-fed colicky infants showed a substantial rise in fecal *Lactobacilli* and a drop in fecal Escherichia (*E.*) *coli* (Savino et al., 2010; Savino et al., 2018b). From these findings, the therapeutic effectiveness of *L. reuteri* could be influenced by the microbiome composition of the infant’s GI tract, which would account for the differences between the effect on crying duration in breast-fed and formula-fed infants.

**Table 1:** Randomized controlled trials of infantile colic.

Year	Intervention	Duration	population	Outcome	Reference
2021	partially hydrolysed formula + maltodextrins+ +DSM 17938 vs standard formula	28 days	Formula-fed colicky infants (<4 months)	significantly lower mean daily crying time in the standard formula group	(Turco et al., 2021)
2018	DSM 17938 vs placebo	1 month	breast-fed colicky infants (<4 months)	significantly shorter crying times, increased circulating FOXP3 concentration, reduced fecal calprotectin in the probiotic group	(Savino et al., 2018b)
2018	DSM 17938 vs placebo	28 days	breast-fed colicky infants (<60 days)	a significant decrease in daily crying time, increased mRNA expression of FoxP3	(Savino et al., 2018a)
2017	DSM 17938 vs placebo	42 days	breast-fed colicky infants (3 weeks to 3 months)	did not significantly change crying time	(Fatheree et al., 2017)
2015	DSM 17938 vs placebo	21 days	breast-fed colicky infants (<60 days)	significantly shorter crying and fussing times in the probiotic group	(Chau et al., 2015)
2015	DSM 17938 and Vit D_3_ vs Vit D_3_	3 months	Newborns (<10 days)	significantly reduced pediatric consultations for infantile colic	(Savino et al., 2015)
2015	DSM 17938 vs placebo	21 days	breast-fed colicky infants (<4 months)	significantly reduced daily crying time, improvement in maternal depression in the probiotic group	(Mi et al., 2015)
2014	DSM 17938 vs placebo	3 months	newborns (<1 week)	significantly reduced duration of crying time	(Indrio et al., 2014)
2014	DSM 17938 vs placebo	1 month	Breast-fed and formula- fed colicky infants (<3 months)	No benefit (increased fussing occurred only in formula fed infants)	(Sung et al., 2014)
2013	DSM 17938 vs placebo	21 days	Breast-fed colicky infants (<5 months)	significantly reduced crying time in the probiotic group	(Szajewska et al., 2013)
2010	DSM 17938 vs placebo	21 days	Breast-fed colicky infants (2 to 16 weeks)	significantly reduced crying time in the probiotic group, a significant increase in fecal lactobacilli and a reduction in fecal Escherichia coli	(Savino et al., 2010)
2007	ATCC 55730 vs simethicone	28 days	Breast-fed colicky infants (21 to 90 days)	significantly reduced daily crying times	(Savino et al., 2007)
2020	DSM 26866 vs placebo	28 days	pregnant women (last 4 weeks of pregnancy)	significantly decreased frequency of colic and lower colic severity in the intervention group	(Pourmirzaiee et al., 2020)

It is also reported that colicky infants showed increased serum concentrations of inflammatory interleukin (IL)-8, monocyte chemoattractant protein-1, and higher fecal calprotectin levels, indicating the existence of systemic and intestinal local inflammation (Partty et al., 2017). *L. reuteri* DSM 17938 treatment can increase circulating FoxP3 concentration and reduce fecal calprotectin, indicating the underlying mechanisms of *L. reuteri* to improve colic may be linked to the property of alleviating inflammation (Savino et al., 2018a; Savino et al., 2018b). According to a recent secondary analysis research based on a large observational study, *L. reuteri*-containing formula was superior to standard formula without probiotics in preventing infant colic, similar to breast feeding (Happy Tummy et al., 2023). Furthermore, the probiotic potential of *L. reuteri* DSM 17938 given at a dosage of 1×10^8^ CFU per day for 90 days to prevent IC is demonstrated by two RCT studies in newborns (Indrio et al., 2014; Savino et al., 2015). This statement was also supported by one prospective cohort study, which showed maternal consumption with *L. reuteri* DSM 26866 in the latter four weeks of gestation was able to protect infants from colic (Pourmirzaiee et al., 2020). In conclusion, since *L. reuteri* may ameliorate colic symptoms mainly by altering the microbial colonization pattern in colicky infants, it is a receptive therapy for colicky infants without side effects and recommendable supplements for newborns to prevent IC.

### 2.2 Diarrhea

Recent data has shown that acute diarrheal disease leads to 179 million outpatient visits every year in the United States, associated with various pathogen infections such as *Shigella*, *E. coli*, rotavirus, norovirus and antibiotic use (Meisenheimer Es Md et al., 2022). Diarrhea is also identified as the leading infectious cause of childhood morbidity and mortality (Ahmed et al., 2021). Previously several specific probiotic strains have demonstrated antidiarrheal effects (Skrzydlo-Radomanska et al., 2020; Lukasik et al., 2022). An increasing number of studies have been conducted to clarify the function of *L. reuteri* in diarrhea since Shornikova and his colleagues first analyzed the effectiveness of *L. reuteri* DSM 17938 in pediatric acute watery diarrhea and demonstrated that it can dosage-dependently shorten the duration of acute watery diarrhea (Shornikova et al., 1997a; Shornikova et al., 1997b). Previous nine RCTs, which demonstrated that *L. reuteri* DSM 17938 was capable of reducing the frequency, length of time, and incidence of diarrhea in children and adults, especially in those with lower nutritional status, supported the drug’s potential benefits in treating and preventing diarrheal diseases (Agustina et al., 2012; Francavilla et al., 2012; Jones et al., 2013; Dinleyici et al., 2014; Gutierrez-Castrellon et al., 2014; Dinleyici et al., 2015; Pernica et al., 2017; Maragkoudaki et al., 2018; Kambale et al., 2023). Additionally, *L. reuteri* DSM 17938 could shorten a child’s stay in the hospital due to acute gastroenteritis or infectious diarrhea (Dinleyici et al., 2014; Szymanski and Szajewska, 2019). However, in the situation of nosocomial diarrhea, three RCTs produced contrary findings (Wanke and Szajewska, 2012; Urbanska et al., 2016; Kolodziej and Szajewska, 2019). The investigators found that *L. reuteri* DSM 17938 was ineffective in preventing diarrhea in inpatient children received antibiotics, regardless of the given dosage (1×10^8^ CFU once daily, or 2×10^8^ CFU twice daily, or 2×10^9^ CFU once daily while in hospital) (Wanke and Szajewska, 2012; Urbanska et al., 2016; Kolodziej and Szajewska, 2019). While *L. reuteri* ATCC 55730 was reported to be able to prevent antibiotic-associated diarrhea in hospitalized adults in another study (Cimperman et al., 2011). Different from the prior studies, this clinical trial analyzed adult patients who were mainly treated with combined antibiotic therapy, and the subjects were given *L. reuteri* ATCC 55730 1×10^8^ CFU twice daily for 28 days (Cimperman et al., 2011). Given these limited data, the function of *L. reuteri* in preventing antibiotic-associated diarrhea is ambiguous, which may be strain-specific and depend on the gut microbiota community disrupted by antibiotics. Of note, none of the mentioned clinical studies analyzed the fecal *L. reuteri* abundance before or after *L. reuteri* administration. All these RCT studies are depicted in **Table 2**.

**Table 2:** Randomized controlled trials of diarrhea and functional constipation.

Year	intervention	symptom	population	Outcome	Reference
2023	DSM 17938+ GG vs placebo for 1 month	diarrhea	Children with uncomplicated severe acute malnutrition	lower number of days of diarrhea lower risk of diarrhea (>16 months)	(Kambale et al., 2023)
2019	DSM 17938 vs placebo for the duration of antibiotic treatment	diarrhea	Hospitalized children received antibiotics	not effective in the prevention of diarrhea	(Kolodziej and Szajewska, 2019)
2019	DSM 17938+ rehydration therapy vs placebo+ rehydration therapy for 5 days	diarrhea	children with acute gastroenteritis (<5 years)	did not reduce the duration of diarrhea a shorter duration of hospitalization	(Szymanski and Szajewska, 2019)
2018	DSM 17938+ oral rehydration salts+ zinc vs placebo + oral rehydration salts+ zinc	diarrhea	non-hospitalized infants with acute diarrhea	without statistical significance but a better trend of the severity and duration of diarrhea	(Maragkoudaki et al., 2018)
2017	DSM 17938 vs placebo for 60 days	diarrhea	hospitalized children with acute diarrhea (2-60 months)	lower odds of recurrent diarrhea	(Pernica et al., 2017)
2016	DSM 17938 vs Placebo	diarrhea	Hospitalized children (1-48 months)	not effective in preventing nosocomial diarrhea	(Urbanska et al., 2016)
2015	DSM 17938 +oral rehydration salts vs oral rehydration salts for 5 days	diarrhea	outpatient children with acute infectious diarrhea	reduced duration of diarrhea	(Dinleyici et al., 2015)
2014	DSM 17938 vs placebo for 5 days	diarrhea	Hospitalized children with acute gastroenteritis	reduced the duration of diarrhea, reduced mean hospital stays	(Dinleyici et al., 2014)
2014	DSM 17938 vs placebo for 3 months	diarrhea	preschool children (6-36 months)	reduced the frequency and duration of episodes of diarrhea	(Gutierrez-Castrellon et al., 2014)
2013	NCIMB 30242 vs Placebo for 9 weeks	diarrhea	healthy hypercholesterolemic subjects	Improved symptoms related to diarrhea	(Jones et al., 2013)
2012	DSM 17938 vs Placebo	diarrhea	Hospitalized children (1-48 months)	no effect on the overall incidence of nosocomial diarrhea	(Wanke and Szajewska, 2012)
2012	DSM 17938 vs Placebo	diarrhea	Healthy children (1-6 years)	reduced diarrhea incidence in children with lower nutritional status	(Agustina et al., 2012)
2012	DSM 17938+oral rehydration salts vs placebo + oral rehydration salts	diarrhea	Hospitalized children with acute diarrhea (6-36 months)	reduced the duration of watery diarrhea	(Francavilla et al., 2012)
2011	ATCC 55730 vs placebo for 4 weeks	diarrhea	hospitalized adults	lower frequency of antibiotic-associated diarrhea	(Cimperman et al., 2011)
2020	DSM 17938 vs placebo	functional constipation	Children with cerebral palsy and chronic constipation	a significant decrease in stool pH and increased defecation, improvement in the history of excessive stool retention, the presence of fecal mass in the rectum, painful defecation,	(Garcia Contreras et al., 2020)
2020	DSM 17938 + magnesium oxide vs DSM 17938+ placebo vs placebo+ magnesium oxide for 4 weeks	functional constipation	Children with functional constipation (6 months -6 years)	significant improvement in defecation frequency in DSM 17938 group	(Kubota et al., 2020)
2019	DSM 17938 vs placebo for 105 days	functional constipation	adults with functional constipation	significantly reduce the serum levels of 5-HT	(Riezzo et al., 2019)
2018	DSM 17938 + macrogol vs placebo + macrogol for 8 weeks	functional constipation	Children with functional constipation	No significant difference	(Wegner et al., 2018)
2018	DSM 17938 vs placebo for 105 days	functional constipation	Adults with functional constipation	helps for defecation, improved abdominal discomfort, pain and bloating	(Riezzo et al., 2018)
2018	DSM 17938 + lactulose vs placebo + lactulose	functional constipation	Children with functional constipation	no difference in the stool frequency, stool consistency, pain	(Jadresin et al., 2018)
2014	DSM 17938 vs placebo for 4 weeks	functional constipation	Adults with functional constipation	increased bowel movements	(Ojetti et al., 2014)
2010	DSM 17938 vs placebo for 8 weeks	functional constipation	Infants with functional constipation (> 6 months)	higher frequency of bowel movements, no significant difference in stool consistency	(Coccorullo et al., 2010)

Enterotoxigenic *E. coli* is a main pathogen of infectious diarrhea in childhood and postweaning piglets (Kotloff, 2022; Qin et al., 2023b). Gut microbiota disruption observed in diarrheal piglets was described as a reduction of *Lactobacillus*, enriched abundance of *E. coli*, and increased lipopolysaccharide (LPS) biosynthesis (Li et al., 2023b). Supplement of *L. reuteri* can reverse the enriched *Pseudomonadota* and the depleted *Bacteroidota* triggered by *E. coli* infection and reduce jejunum *E. coli* content, thus preserving intestinal disruption (Wang et al., 2018; Lu et al., 2023). *L. reuteri* stains including *L. reuteri* HCM2 and *L. reuteri* PSC102 have been reported to suppress the growth of *E. coli in vitro* and inhibit its adherence to intestinal epithelial cells (Wang et al., 2018; Ali et al., 2023). Moreover, *L. reuteri* was also reported to possess the ability to upregulate the expression of tight junction (TJ) proteins and E-cadherin, thereby maintaining intestinal permeability in infected mice (Karimi et al., 2018; Lu et al., 2023). In a rotavirus-infected murine model, *L. reuteri* can reduce diarrhea duration and alleviate inflammation, probably associated with accelerated intestinal epithelium turnover and restored gut microbiome diversity (Preidis et al., 2012). Particularly, the efficiency of *L. reuteri* to increase enterocyte proliferation and migration seemed to be influenced by host nutritional status (Preidis et al., 2012). Except these, the therapeutic application of *L. reuteri* in infectious diarrhea may also be related to its metabolite production such as reuterin, with the ability to inhibit an extensive range of germs (Collinson et al., 2020).

These results illustrated that the therapeutic efficiency of *L. reuteri* in diarrhea may depend on regulation of gut microbiota and barrier, which is strain-specific and reliant on age, pathogen, nutritional status, and the disease situation as well. To sum up, *L. reuteri* can be recommended as an adjuvant to rehydration treatment for diarrhea in therapeutic settings, but its function in prophylactic settings still requires more research, especially in the antibiotics-use setting.

### 2.3 Constipation

Constipation is an intestinal condition that could affect individuals of all ages and dramatically lower their quality of life (Saviano et al., 2021). With a prevalence spanning from 7% to 30%, constipation is also one of the most prevalent pediatric disorders (Wegner et al., 2018). The fundamental mechanism of constipation, which typically has a functional cause without an organic origin, is largely unknown (Mulhem et al., 2022). It is suggested that disruption of the intestinal flora play a role in functional constipation (FC) (Zhang et al., 2021). The results may be conflict reliant on measurements. Based on traditional microbiological culture tests, enriched *Bifidobacteria* and *Lactobacillus* were observed in children with FC, while decreased abundance of *Bifidobacterium* and *Lac*tobacillus was seen in adult FC patients (Zoppi et al., 1998; Khalif et al., 2005). Nowadays though gene sequencing analysis, it seems consistent that *Firmicutes* relative abundance is increased while *Bacteroidetes* frequency is heterogeneous based on population, and the gut microbe pattern differs in different subtypes of constipation as well (Zhu et al., 2014; Mancabelli et al., 2017; Yu et al., 2023a).

A couple of RCTs have evaluated the effectiveness of *L. reuteri* DSM 17938 in treating FC, depicted in **Table 2**. The investigations demonstrated that *L. reuteri* DSM 17938 single-drug administration significantly alleviated constipation both in children and adults, involving improvement in defecation frequency, painful defecation, and reduced fecal mass in the rectum (Coccorullo et al., 2010; Ojetti et al., 2014; Riezzo et al., 2018; Garcia Contreras et al., 2020; Kubota et al., 2020). However, *L. reuteri* DSM 17938 did not show additional improvement in FC when regarded as adjuvant therapy for lactulose or macrogol (Jadresin et al., 2018; Wegner et al., 2018; Kubota et al., 2020). Only one study investigated the gut microbiome community alternations, in which the limited data revealed that *L. reuteri* treatment did not alter the gut microbiota composition of the pediatric subjects with FC (Kubota et al., 2020).

To note, current evidence showed that *L. reuteri* DSM 17938 improved bowel movements, but did not affect stool consistency (Coccorullo et al., 2010; Kubota et al., 2020). The defecation frequency was found to be negatively correlated with genus *Oscillospira*, *Megasphaera*, and *Ruminococcus* (Kubota et al., 2020). On one hand, *L. reuteri* DSM 17938 can improve intestinal motility and promote intestinal transit, partially via controlling some GI peptide pathways. According to research by Riezzo and colleagues, the therapeutic efficacy of *L. reuteri* DSM 17938 in FC may be associated with serum brain-derived neurotrophic factor and serotonin (5-HT) concentrations (Riezzo et al., 2019). Tryptophan metabolism may be impacted by *L. reuteri*, which would reduce the level of 5-HT in circulation (Montgomery et al., 2022). The connection between the intestinal flora and the enteric neural systems involves the 5-HT pathways, which have advantages for irritating the local enteric nerve responses and promoting motility (Ge et al., 2017; Agus et al., 2018). On the other hand, *L. reuteri* DSM 17938 is capable of producing short-chain fatty acids (SCFAs), the substances involved in boosting intestinal peristalsis and colonic myoelectric cell response, all of which are effective for treating chronic FC (Wu et al., 2013; Hurst et al., 2014; Calvigioni et al., 2023).

However, there is still no sufficient data in the field of *L. reuteri* application for constipation prevention. A prospective RCT study carried out by Indrio and colleagues revealed that oral supplementation with *L. reuteri* DSM 1793 in newborns effectively prevented constipation within the first 3 months after birth (Indrio et al., 2014). In conclusion, present findings indicate that *L. reuteri* can be recommended to be used in the management of FC as an alternative therapy for traditional drugs.

### 2.4 Functional abdominal pain

FAP, which is the most prevalent type of abdominal pain in children and teenagers, with a prevalence of about 14 percent, is described as chronic or recurring pain without an organic cause (Bao et al., 2023). Although most FAP is often self-managed, it can have adverse effects on life quality, suffered children’s after-school activities, and school attendance, and may interfere with regular family life (Thapar et al., 2020). The disease was currently considered a disorder of the “brain-gut axis”, and gut microbiome changes could be engaged in its pathogenesis, but the available data is insufficient to draw a conclusion (Zhou et al., 2022a). Given the limitations of medication in children, probiotics arise as a possibly pleasant therapeutic approach for the treatment of pediatric FAP (Trivic et al., 2021). Six RCT investigations on *L. reuteri* DSM 17938’s medicinal potential are summarized in **Table 3**. The studies demonstrated that administration of *L. reuteri* DSM 17938 for 4 to 12 weeks reduced the pain intensity, the frequency of episodes, and increased days without pain in irritable bowel syndrome or FAP-affected children without adverse events (Romano et al., 2014; Eftekhari et al., 2015; Weizman et al., 2016; Jadresin et al., 2017; Maragkoudaki et al., 2017; Jadresin et al., 2020). However, one study proposed that *L. reuteri* DSM 17938 was equally effective in relieving pain in both the placebo and *L. reuteri* groups of children with FAP (Eftekhari et al., 2015). The authors speculated that the result may be explained by psychological effects since *L. reuteri* DSM 17938 showed no superiority to placebo (Eftekhari et al., 2015). None of the studies explored gut flora profiles after *L. reuteri* treatment, so we cannot draw a conclusion about the precise mechanism of *L. reuteri* application in pediatric FAP.

**Table 3:** Randomized controlled trials of functional abdominal pain.

Year	intervention	population	Outcome	References
2020	DSM 17938 vs placebo for 12 weeks	Children with functional abdominal pain (4-18 years)	significant increased days without pain, reduced intensity of pain (follow up of 4weeks)	(Jadresin et al., 2020)
2017	DSM 17938 vs placebo for 12 weeks	Children with functional abdominal pain or irritable bowel syndrome (4-18 years)	significantly reduced severity of abdominal pain, increased days without pain (follow-up of 4weeks)	(Jadresin et al., 2017)
2017	DSM 17938 vs placebo for 4 weeks	Children with functional abdominal pain (5-16 years)	reduced the frequency and intensity of abdominal pain episodes (follow-up of 8weeks)	(Maragkoudaki et al., 2017)
2016	DSM 17938 vs placebo for 4 weeks	Children with functional abdominal pain (6-15 years)	relieving frequency and intensity of functional abdominal pain (follow-up of 4weeks)	(Weizman et al., 2016)
2015	DSM 17938 vs placebo for 4 weeks	Children with functional abdominal pain (4-16 years)	the pain was significantly reduced in both groups, but no significant difference compared with placebo (follow-up of 8weeks)	(Eftekhari et al., 2015)
2014	DSM 17938 vs placebo for 4 weeks	Children with functional abdominal pain (6-16 years)	lower pain intensity (follow-up of 4 weeks)	(Romano et al., 2014)

By enhancing opioid receptor activity in the afflicted tissues, Hegde and his colleagues reported that the dorsal root ganglia neurons projecting to the dilated colon exhibited attenuated hyperexcitability after *L. reuteri* recolonization, thus preventing visceral hypersensitivity in lumen distension in rats (Hegde et al., 2020). Additionally, *L. reuteri* may exhibit the ability to reduce gut pain by reducing signaling from the pain receptor transient receptor potential vanilloid 1 channel in the intestine (Pang et al., 2022). Collectively, although *L. reuteri* could be an attractive choice in treating FAP-related diseases due to its safety feature, there is currently no powerful proof to recommend *L. reuteri* application in FAP and further long-run researches are still needed to explore the definite mechanism.

### 2.5 Helicobacter pylori infection

*Helicobacter pylori* (*H. pylori*) is the key pathogen of chronic active gastritis, peptic and duodenal ulcers, with the global prevalence of infection exceeding 50%, also considered a high-risk factor for gastric cancer (de Brito et al., 2019). Currently, the effectiveness of treatment has been greatly impaired by the fast global emergence of antibiotic resistance to *H. pylori*, and the quadruple approach for *H. pylori* elimination could further exacerbate GI disease (Chen et al., 2021). It is suggested that eradication of *H. pylori* may lead to disruption of gut microbiota, featured by reduced *Actinobacteria* as well as *Bacteroidetes* abundances and enriched *Proteobacteria* (Sitkin et al., 2022). Specific probiotics may be helpful in reducing side effects triggered by first-line antimicrobial therapy and enhancing the efficacy of *H. pylori* eradication therapy (Liang et al., 2022). In previous RCT studies, *L. reuteri* was shown to reduce *H. pylori* load but seemed to be unable to increase eradication rates of *H. pylori* as an adjuvant for first-line therapy (Imase et al., 2007; Francavilla et al., 2008; Mehling and Busjahn, 2013; Francavilla et al., 2014; Holz et al., 2015; Dore et al., 2019; Poonyam et al., 2019; Yang et al., 2021; Dore et al., 2022; Moreno Marquez et al., 2022; Naghibzadeh et al., 2022), shown in **Table 4**. Notably, several studies showed that *L. reuteri* can reduce abdominal pain, distension, and other eradication treatment-related adverse events, consistent with decreased GI symptom rating scale scores in *H. pylori*-positive subjects, thus governing patient compliance (Lionetti et al., 2006; Francavilla et al., 2008; Francavilla et al., 2014; Poonyam et al., 2019; Yang et al., 2021; Moreno Marquez et al., 2022). Two studies analyzed the alternations of the gut flora after combined therapy of this *probiotics* and eradication treatment, which revealed that *L. reuteri* can change the gut microbiota composition in *H. pylori*-positive patients but cannot fully counteract gut dysbiosis induced by antibiotics (Yang et al., 2021; Dore et al., 2022). It means that the benefits brought by *L. reuteri* supplement cannot be attributed to restored gut microbiota balance. Particularly, *L. reuteri* can survive the gastric acid environment and colonize the gastric mucosa (Dargenio et al., 2021). It is reported that *L. reuteri* can inhibit the early colonization stages of *H. pylori* in the human GI tract, associated with the suppression of the binding of *H. pylori* to putative glycolipid receptor molecules (Holz et al., 2015). Besides, *L. reuteri* produces reuterin, an antibiotic that targets *H. pylori*, thus reducing *H. pylori* load (Urrutia-Baca et al., 2018). Meanwhile, a most recent survey also revealed that *L. reuteri* 2892 attenuated *H. pylori*-induced gastritis by its anti-inflammatory and anti-oxidative stress characteristics as well as suppressing the gene expression of the virulence factor CagA (Forooghi Nia et al., 2023). Recent studies also focus on the relationship between *L. reuteri* and cancer diseases, it has been demonstrated that *L. reuteri* exerts an anti-tumor effect in gastric cancer MKN1 cells, but data is still limited about *L. reuteri* application in gastric cancers related to *H. pylori* infection (Kim et al., 2022a).

**Table 4:** Randomized controlled trials of *H. pylori* infection.

Year	intervention	population	Outcome	References
2022	quadruple therapy+ S. boulardii vs quadruple therapy+ DSMZ 17648 vs quadruple therapy	H. pylori-positive adults	no significant difference in the eradication of H. pylori in *L. reuteri* group	(Naghibzadeh et al., 2022)
2022	quadruple therapy + DSM 17938 and ATCC PTA 6475 vs quadruple therapy	H. pylori-positive adults	no significant difference in the eradication of H. pylori	(Dore et al., 2022)
2022	eradication regimen+ DSM 17938 and ATCC 6475 vs eradication regimen	H. pylori-positive adults	no differences in eradication therapy, reduced abdominal pain and distension	(Moreno Marquez et al., 2022)
2021	non-viable DSM17648+triple therapy vs placebo + triple therapy	H. pylori-positive adults	did not improve the eradication rate of H. pylori, reduced abdominal distention, diarrhea, and the Gastrointestinal Symptom Rating Scale score.	(Yang et al., 2021)
2019	quadruple therapy+ Biogaia^®^ vs quadruple therapy	H. pylori-positive adults	did not increase eradication rates, reduced treatment-related adverse events and improve the patients’ compliance	(Poonyam et al., 2019)
2019	quadruple therapy + DSM 17938 and ATC 6475 vs quadruple therapy	H. pylori-positive adults	no significance difference in H. pylori-eradication rate	(Dore et al., 2019)
2015	non-viable DSM17648 vs placebo	H. pylori-positive adults	reduce the load of H. pylori	(Holz et al., 2015)
2014	eradication regimen+ DSM 17938 and ATCC 6475 vs eradication regimen	H. pylori-positive adults	reduced load of H. pylori, fewer side effects, decreased Gastrointestinal Symptom Rating Scale scores, no difference in the H. pylori-eradication rate	(Francavilla et al., 2014)
2013	non-viable DSMZ17648 vs placebo	H. pylori-positive asymptomatic adults	reduced load of H. pylori	(Mehling and Busjahn, 2013)
2008	ATCC 55730+ sequential conventional treatment vs sequential conventional treatment	H. pylori-positive adults	reduced H. pylori load, decreased Gastrointestinal Symptom Rating Scale scores, no difference in eradication rates	(Francavilla et al., 2008)
2007	SD2112 vs placebo	H. pylori-positive adults	reduced load of H. pylori	(Imase et al., 2007)
2006	sequential therapy + ATCC 55730 vs sequential therapy	H. pylori-positive children	reduced frequency and intensity of antibiotic-associated side effects, decreased Gastrointestinal Symptom Rating Scale scores	(Lionetti et al., 2006)

Overall, *L. reuteri* has shown superiority as a salvageable treatment for side effects brought by eradication therapy, yet we still need further studies to delve deeper into the research gap between *L. reuteri* application and *H. pylori*-associated cancer.

### 2.6 Inflammatory bowel disease and diverticulitis

Crohn’s disease and ulcerative colitis are categorized as chronic IBD, which have evolved into a worldwide burden with increasing incidence (Nishikawa et al., 2021). It is reported that about one in five children needing a colectomy during childhood while the intestinal damage and complications brought by Crohn’s disease are still a difficulty for disease management (Cheng et al., 2021; Gordon et al., 2022). Multiple factors, such as the interaction between genetic background and environment, especially including a potential association between impaired gut microbial homeostasis and inflammation, and gut barrier disruption as well, have been suggested to be engaged in IBD pathogenesis (Larabi et al., 2020). It was observed in IBD subjects that the relative abundance of *Proteobacteria*, especially *E. coli*, was increased while the content of *Bacteroidetes* and *Firmicutes* was depleted, along with the reduced gut microbiota diversity (Nishino et al., 2018; Vester-Andersen et al., 2019). It has been revealed in a previous clinical trial, whereby, rectal infusion of *L. reuteri* ATCC 55730 effectively improved inflamed mucous membranes and reduced mucosal expression levels of inflammatory markers in active distal ulcerative colitis in children (Oliva et al., 2012), shown in **Table 5**.

**Table 5:** Randomized controlled trials of colitis and diverticulitis.

Year	intervention	symptom	population	Outcome	References
2022	ATCC PTA 4659+ fluids+bowel rest vs placebo+ fluids+bowel rest	abdominal pain, inflammatory markers and reduction of hours of hospitalization.	patients affected by acute uncomplicated diverticulitis	Decreased inflammatory markers	(Ojetti et al., 2022)
2019	ATCC PTA 4659+ ciprofloxacin+metronidazole vs placebo+ciprofloxacin+metronidazole	abdominal pain, inflammatory markers and reduction of hours of hospitalization.	patients affected by acute uncomplicated diverticulitis	reduced abdominal pain and inflammatory markers	(Petruzziello et al., 2019)
2012	ATCC 55730+mesalazine vs placebo+mesalazine	inflammation and cytokine expression of rectal mucosa	in children with active distal ulcerative colitis	decreased mucosal inflammation	(Oliva et al., 2012)

According to Liu and his colleagues, peroral treatment with *L. reuteri* ATCC PTA 4659 improved colitis severity clinically and morphologically in mouse colitis caused by dextran sulfate sodium (Liu et al., 2022a). Other studies investigated the efficiency of *L. reuteri* R2LC, ATCC PTA 4659, F-9-35, and 5454, these strains also can alleviate inflammation in mice colitis, characterized by downregulated pro-inflammatory tumor necrosis factor-α (TNF-α), IL-1β, and interferon-γ, the decisive cytokines in colitis development (Ahl et al., 2016; Sun et al., 2018; Hrdy et al., 2020). This may be explained by the boosted expression of the TJ proteins along with cytoprotective heat shock proteins after *L. reuteri* therapy, thereby conferring protection against defects in gut barrier function (Ahl et al., 2016; Liu et al., 2022a). Meanwhile, *L. reuteri* supplement can also reverse gut microbiota dysbiosis induced by colitis. It was reported that after *L. reuteri* FYNDL13 treatment, enriched beneficial bacteria (*Bifidobacterium*, *Akkermansia*, *Blautia* and *Oscillospira*) together with reduced harmful bacteria (*Bacteroide*s and *Sutterella*) content was observed (Lin et al., 2023). Moreover, *L. reuteri* therapy can offset bacterial translocation to the mesenteric lymph nodes caused by colitis, leading to the amelioration of excessive immune reactions (Liu et al., 2022a). Secretory immunoglobulin (Ig) A is responsible for bacterial translocation and bacterial toxins neutralization in the intestinal mucosa (Tezuka and Ohteki, 2019). Recently, it was discovered that orally administered *L. reuteri* R2LC stimulated the proliferation of the B lymphocytes in Peyer’s patches and induced IgA production and secretion to the intestinal lumen via the PD-1 pathway (Liu et al., 2021a). Consequently, treatment based on *L. reuteri* R2LC regulated alterations in the gut microbiome and shielded against colitis caused by dextran-sulfate-sodium (Liu et al., 2021a). Dendritic cells (DCs) are responsible for handling pathogens when the gut barriers are disrupted in IBD (Wei et al., 2022). *L. reuteri* was proven to possess the potential to promote DCs differentiation and maturation and boost regulatory T cells (Tregs) induction (Haileselassie et al., 2016; Hrdy et al., 2020). In addition, it is demonstrated that *L. reuteri* may reduce intestine inflammation by the mechanisms dependent on aryl hydrocarbon receptor activation, an immune and bacteria sensor receptor, correlated to inflammatory responses in IBD pathogenesis (Pernomian et al., 2020). The tryptophan metabolites from *L. reuteri* can activate aryl hydrocarbon receptors, inducing IL-22 production to maintain mucosal protection (Zelante et al., 2013). And the microbial histamine produced by *L. reuteri* has been proven to ameliorate intestinal inflammation in mice via activating H2R signaling (Gao et al., 2015). It was also reported that LPxTG-motif surface protein derived from *L. reuteri* SH 23 can alleviate inflammation in a mice colitis model via the inhibition of the MAPK-dependent NF-κB transduction way (Zong et al., 2023).

Colon mucosa and submucosa may inflame and herniate through the muscular layer in cases of diverticulitis (Barbaro et al., 2022). Diet and lifestyle factors may induce changes in the gut microbiota community that contributes to mucosal inflammation and diverticulitis (Piccioni et al., 2021). Probiotics could be beneficial in diverticular disease because they can prevent harmful bacteria from adhering to the intestinal mucosa and from producing inflammatory markers (Piccioni et al., 2021). Two previous RCTs examined *L. reuteri*’s function in managing acute uncomplicated diverticulitis (AUD) in light of recent recommendations that the condition be treated without the use of antibiotics (Petruzziello et al., 2019; Ojetti et al., 2022). They revealed that the treatment with *L. reuteri* ATCC PTA 4659 can alleviate abdominal pain and reduce inflammatory markers as well as the duration of hospitalization in patients with AUD (Petruzziello et al., 2019; Ojetti et al., 2022), shown in **Table 5**.

Therefore, the above preclinical studies validate the therapeutic potential of specific strains of *L. reuteri* to enhance the natural defense of gut epitheliums, alleviate hyperimmune response and restore gut microecology balance in inflammatory intestinal diseases. Although clinical data are limited, *L. reuteri* can be an attractive adjuvant therapy for IBD and diverticulitis.

### 2.7 Colorectal cancer

As per global statistics, CRC is ranked third as the most prevalent type of malignancy and the fourth major contributor to deaths related to cancer worldwide (Baidoun et al., 2021). As is known to all, IBD is linked to the onset and progression of CRC (Mathey et al., 2021). Dysplasia, clonal proliferation, and malignant progression might all be caused by gut dysbacteriosis, which may also have dramatic effects on genetics and epigenetics (Eslami et al., 2019). Previously, researchers identified gut flora dysbiosis in patients with CRC, including the depletion of *L. gallinarum*, *Streptococcus thermophilus* and enriched *Firmicutes* and *Bacteroidetes*, along with decreased bacterial diversity (Dai et al., 2018). Reduced abundance of *Lactobacillus, Roseburia* and *Bifidobacterium* in gut microbiota composition was observed in early precursors of CRC including adenoma and serrated polyp (Rezasoltani et al., 2018).

Although the prognosis of CRC patients without local or distant metastatic disease is generally favorable, there are currently inadequate viable therapies for individuals with metastatic cancer (Bell et al., 2022). Accordingly, probiotic therapy has recently gained popularity as a promising CRC therapeutic approach. Gao and colleagues demonstrated that treatment with (histidine decarboxylase) HDC^+^ *L. reuteri* can induce luminal histamine production, consequently, decreased colon tumor frequency and size were observed in HDC^-/-^ mice (Gao et al., 2017). Moreover, they discovered that an *L. reuteri* mutant with isogenic HDC deficiency that cannot generate histamine did not inhibit tumorigenesis, highlighting the essential function of histamine in preventing inflammatory responses and the development of colorectal tumors (Gao et al., 2017). Reuterin, another major compound produced by *L. reuteri*, was found to be downregulated in mice and human CRC (Bell et al., 2022). Consistently, reuterin has been shown to have an inhibitory impact on the proliferative rate of CRC cell lines such as RKO at a concentration of 25µM, but not at a higher dosage of 100µM on normal colonic epithelium (Yu et al., 2023b). Indole-3-lactic acid, a tryptophan catabolite that inhibits the IL-17 signaling pathway, was shown in another recent study to be a key player in the suppression of colorectal carcinogenesis following *L. reuteri* treatment (Han et al., 2023). RAR-related orphan receptor γt, a nuclear receptor, was the target of this microbial metabolite product, which prevented Th17 cell development (Han et al., 2023). The potential underlying mechanism of *L. reuteri*’s pro-apoptotic actions was investigated but still needs further investigation. *L. reuteri* MG5346 was reported to inhibit tumor growth by inducing cell apoptosis though upregulated caspase-9 activity (Kim et al., 2022b). Additionally, it has been demonstrated that sirtuin-3–*L. reuteri* interaction is essential for the development of gut tumors (Zhang et al., 2018). Sirtuin-3 is a tumor-suppressing gene and *L. reuteri* can inhibit the downregulation of *Sirt3* expression during colorectal tumorigenesis (Zhang et al., 2018). A previous meta-analysis analyzed the relationship between intestine E-cadherin and the prognosis of CRC, they concluded that low expression of E-cadherin is a risk factor for poor prognosis in CRC sufferers (Chang et al., 2022). It has been evidenced that *L. reuteri* can boost E-cadherin expression, indicating a possible mechanism for treating CRC (Karimi et al., 2018).

In summary, considering these evidence, *L. reuteri* owns the ability to suppress intestinal tumor progression in direct and indirect ways, thereby it may develop into a potential adjuvant treatment, but it is premature to recommend widespread use of *L. reuteri* in CRC due to insufficient clinical data.

### 2.8 Liver diseases

The total cirrhosis incidence is growing and estimated to reach 112.1/100,000 person-years in 2040, largely attributed to the increase in non-alcoholic fatty liver disease and alcohol-related liver disease (Flemming et al., 2021). The liver is a central immunological organ, which is frequently exposed to metabolites derived from the intestinal microbiome, thus it is not surprising that the GI microbial community also plays a part in the pathologic process of liver diseases (Jones and Neish, 2021; Wang et al., 2021a). Particularly, gut microbiota dysbiosis may already exist at the early period of liver damage. Studies have shown alcohol-dependent subjects with liver disorder show compositional changes in the fecal microbiota, presented as a reduction in bacterial diversity and decreased relative abundance of *Lactobacillus*, *Bifidobacterium*, as well as *Akkermansia muciniphila* (Leclercq et al., 2014; Grander et al., 2018). The phenotype of ethanol-elicited hepatitis was reversed by *L. reuteri*, partially by regulating fatty acid metabolic pathways in mice alcoholic liver disease models (Zheng et al., 2020). It was also reported that *L. reuteri* MJM60668 supplementation to mice fed a high-fat diet ameliorated hepatic steatosis by inhibiting lipogenesis, which may be attributed to the improvement of the gut microbiome dysbiosis (Werlinger et al., 2022). Chen and colleagues reported that gut microbiota imbalance promoted liver tumorigenesis and administration with *L. reuteri* can improve tryptophan metabolism and reduce hepatic sterol regulatory element-binding protein 2 expression, thus suppressing tumorigenesis (Chen et al., 2022). *L. reuteri* was also reported to reduce IL-17A secretion of group 3 innate lymphoid cells (ILC3s) in liver by upregulating acetate levels, thus exerting anti-tumor effects in mice with hepatocellular carcinoma (Hu et al., 2023). These evidences revealed the interaction between gut microbiome and hepatic immune response as well as metabolite signal pathways. Hence the use of *L. reuteri* in managing liver disorders has drawn a lot of attention from researchers, although RCT studies on the bacteria’s protective effects for patients with hepatic ailments are still inadequate. In one RCT research, Ferolla et al. discovered that a 3-months supplementation of *L. reuteri* with guar gum as well as inulin reduced hepatic steatosis in subjects with nonalcoholic steatohepatitis (Ferolla et al., 2016). However, another RCT study carried out in 2017 showed that in obese patients with type 2 diabetes, oral dosing of *L. reuteri* DSM 17938 for 12 weeks seemed to have no impact on the microbiota composition, adiposity, or liver steatosis, although, in some subjects with higher gut microbiota diversity before intervention, it increased insulin sensitivity (Mobini et al., 2017). The contrary outcomes may be attributed to the of microbial diversity in complicated metabolic disease conditions and the crosstalk caused by obesity. Taken together, these preclinical literatures provide evidence that *L. reuteri* may relieve liver injury in several signaling pathways probably by reversing gut microbiota dysbiosis. Given this, it may act as a potential probiotic for the prevention or therapeutic strategy for liver diseases, but further long-term well-designed clinical trials are still needed.

## 3 Potential *L. reuteri* mechanisms in digestive system diseases

**Figure 1:**
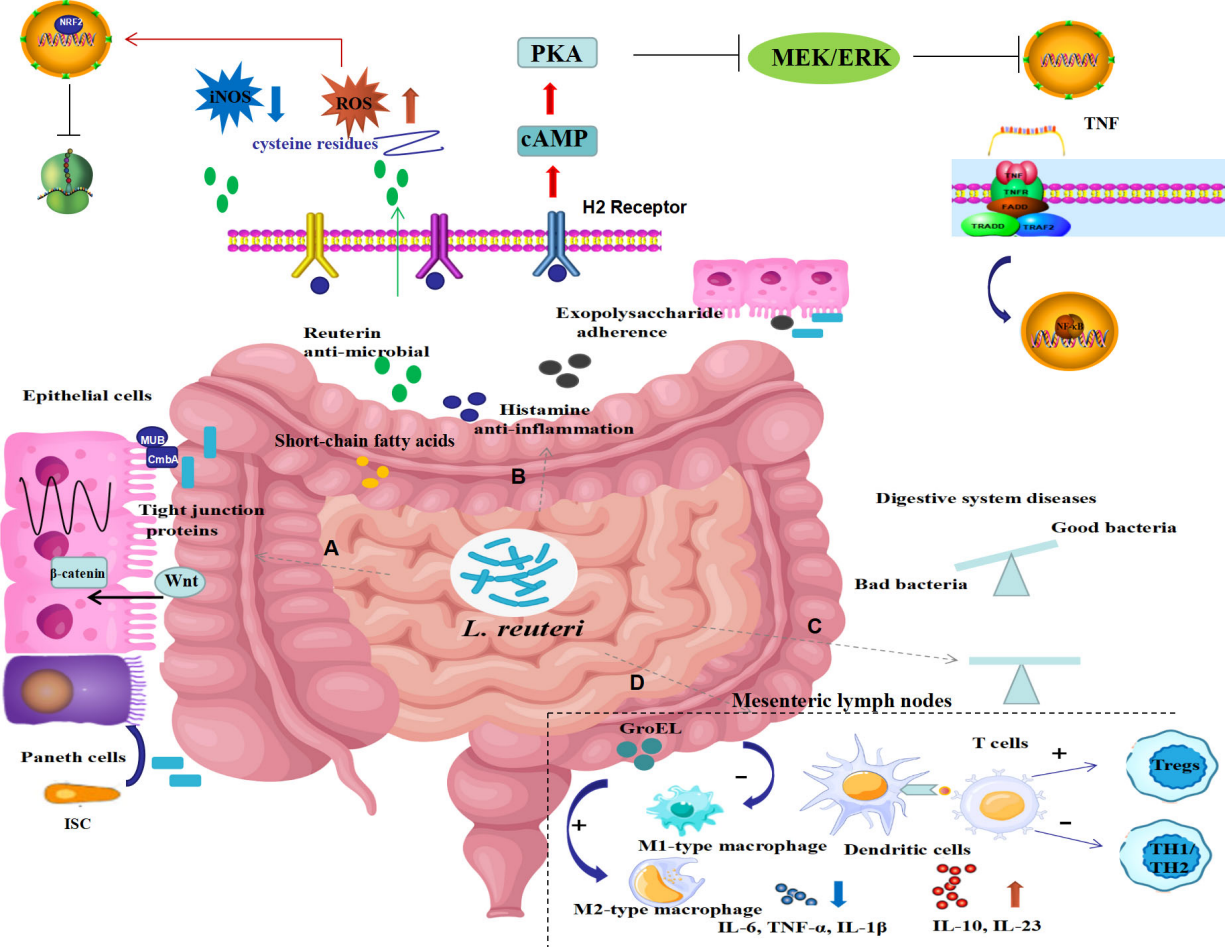
Possible underlying mechanisms of *L. reuteri* in digestive system diseases. The underlying mechanisms of *L. reuteri* application in intestinal diseases may include the following ways: **(A)** preservation of gut barrier function by increasing expression of tight junction proteins, promoting the intestine epithelial cell proliferation, and inducing intestinal stem cell differentiation to Paneth cells by activating Wnt/β-catenin pathway; **(B)** production of metabolites including reuterin, histamine, exopolysaccharide, short-chain fatty acids, thus mediating antibacterial, anti-inflammatory and anti-oxidative stress properties via inhibited NF-κB signaling pathways as well as activated NRF2 signaling pathways; **(C)** modification of the composition of the gut bacteria to restore balance; **(D)** regulation of intestinal immune response by selecting macrophage phenotype, promoting dendritic cell differentiation, suppressing Th1/Th2 responses, inducing proliferation of regulatory T cells, thereby suppressing inflammatory responses. ISC, intestine epithelial cell; ROS, reactive oxygen species; iNOS, inducible nitric oxide synthase; cAMP, cyclic adenosine monophosphate; PKA, Protein kinase A; TNF, tumor necrosis factor; NRF2, nuclear factor erythroid-derived 2; H2 receptor, histamine 2 receptor; IL, interleukin; Tregs, regulatory T cells.

As illustrated in **Figure 1**, growing preclinical investigations revealed that *L. reuteri* restores gut microbiota balance, products antimicrobial metabolites, regulates intestinal immunity and mediates mucosal homeostasis, thereby exhibiting protective effects on digestive system diseases.

### 3.1 Remodeling the gut microbiota

It is well-documented that *L. reuteri* can alter the bacterial diversity and abundance of various microorganisms, largely strain-specific. For instance, administration with *L. reuteri* LR6 and KT260178 both resulted in higher counts of total *Lactobacillus*, *Bifidobacterium* (Garg et al., 2020; Yang et al., 2020). While *L. reuteri* DSM 17938 supplement improved bacterial diversity along with enriched *Firmicutes* and reduced abundance of *Bacteroidetes* (Liu et al., 2019). Similar results have been observed in disease conditions. After supplement with *L. reuteri* DSM 17938, investigators observed enriched fecal *Lactobacilli* and a decrease in the colicky infants’ fecal *E. coli* counts (Savino et al., 2010; Savino et al., 2018b). Consistently, *L. reuteri* ATCC PTA 4659 peroral therapy alleviated mice colitis by maintaining the diversity of the colon’s microbiome (Liu et al., 2022a).

The beneficial properties of *L. reuteri* on gut microflora dysbiosis may be partially explained by the competition of *L. reuteri* and these harmful genera for the intestinal epithelium binding sites (Walsham et al., 2016). Walsham et al. reported that *L. reuteri* can bind to the epithelial cell surface by mucus-binding proteins CmbA and MUB, thus inhibiting enteropathogenic *E. coli* adherence to the gut epithelium (Walsham et al., 2016). Bioinformatics analysis further revealed that the inhibition mechanism may be associated with PI3K-Akt and MAPK signaling pathways (Qin et al., 2023b). Furthermore, *L. reuteri* may inhibit harmful bacteria by the production of antimicrobial metabolites like reuterin (Asare et al., 2020). The probiotic management of the gastrointestinal microbiota may also be mediated by regulating the intestinal pH through lactic acid production as well as some other antimicrobial substances like acetic acid and ethanol of some *L. reuteri* strains (Vitale et al., 2023). *L. reuteri* is demonstrated to be effective against a range of GI microbial infections, including *Salmonella*, *Enterobacteriaceae*, and others, partially depending on the synthesis of these compounds (Kao et al., 2020; Laosee et al., 2022; Qin et al., 2023a). In mice with colitis caused by dextran-sulfate-sodium, dosing of *L. reuteri* R2LC may remodel the makeup of the gut microbiome via enhanced intestinal IgA production (Liu et al., 2021a). It has been demonstrated that IgA is responsible for the selection of intestinal colonized bacteria and tends to resist pathogenic bacteria (Schnupf et al., 2018).

Overall, *L. reuteri* is shown to modulate the gut microbial community, characterized by enriched beneficial genera and diminished pathogenetic genera. Nonetheless, further research is required to determine the interaction between GI health and the modulated gut microbiota.

### 3.2 Production of antimicrobial metabolites

With the development of metabolomics and bacterial genetics strategy, small compounds produced by *L. reuteri* were identified. It has been proposed that the metabolite products of *L. reuteri* strains are responsible for their antibacterial and immunoregulatory properties. We covered a few well-known metabolites associated with *L. reuteri*’s probiotic potential in this section.

#### 3.2.1 Reuterin

Reuterin is a broad-spectrum antimicrobial compound generated by specific strains of *L. reuteri* during the anaerobic metabolism of glycerol (Asare et al., 2020). This antimicrobial compound, which is a blend of different 3-hydroxypropionaldehyde (3-HPA) forms, may suppress the proliferation of both Gram-positive and -negative bacteria, protozoa, and fungi (Asare et al., 2020; Saviano et al., 2021). Apart from being used as a food preservative product, reuterin was also identified as the most inhibitory compound in CRC cell lines (Bell et al., 2022). Recently, it was shown that reuterin can suppress tumor growth in the mouse model implanted with CRC xenograft tumors by inducing oxidative stress (Bell et al., 2022). The authors showed that reuterin can inhibit the growth of CRC cell lines with a dose of 25μM but the concentration of reuterin at a dose of 100μM seemed not cytotoxic to non-cancerous colon cell lines (Bell et al., 2022). The molecular mechanism might be explained by that reuterin can increase reactive oxygen species and thus reduce ribosomal biogenesis by selectively binding to cysteine residues, accompanied by increased gene expression downstream of nuclear factor erythroid-derived 2 (Nrf-2) (Bell et al., 2022). However, it was also revealed *in vitro* experiments that reuterin treatment at a dose of 250μM significantly suppressed LPS-induced oxidative stress by enhancing Nrf-2 expression and reducing reactive oxygen species production in HD11 macrophage cells (Xu et al., 2022). These data revealed that the effects of reuterin depend on certain cell types, reuterin concentration, and stimulation time.

#### 3.2.2 Histamine

Biogenic amines, especially histamine, were reported to be able to modulate the host immune system (Dvornikova et al., 2023). Several strains of *L. reuteri* such as *L. reuteri* DSM 20016 are capable of producing histamine from the dietary component L-histidine, an amino acid (Smythe and Efthimiou, 2022). According to Lin and his colleagues, *L. reuteri* ATCC PTA 6475 inhibited the MAPK signaling pathway, reducing the generation of TNF in monocyte-derived macrophages isolated from children with Crohn’s disease (Lin et al., 2008). By using mass spectrometry, investigators further proved that the key substance of *L. reuteri* ATCC PTA 6475-mediated TNF inhibition was histamine (Thomas et al., 2012). Histamine’s pleiotropic actions are achieved by activating the H1R, H2R, H3R, and H4R histamine receptors present in mammalian cells (Shulpekova et al., 2021). Via activation of H2R on human monocytoid cell line THP-1 cells, histamine stimulated increased levels of cyclic adenosine monophosphate (cAMP) and improved protein kinase A activity, thus inhibiting MEK/ERK MAPK signaling and alleviating inflammation (Thomas et al., 2012). Consistently, Gao and his colleagues discovered that oral administration of HDC^+^ *L. reuteri* capable of converting L-histidine to histamine might successfully alleviate inflammatory reactions in a murine model of colitis (Gao et al., 2015). Subsequently, they found that by causing immature myeloid cells to aggregate, HDC deficiency can accelerate the development of CRC that is linked to inflammation, hinting that histamine may have antitumorigenic properties (Gao et al., 2017). H1R, H2R, and, to a less extent, H4R are expressed by myeloid cells in the GI tract (Sander et al., 2006). Consequently, histamine may be involved in intestinal immunomodulation and inflammation by mediating myeloid cells (Sander et al., 2006). Besides, it is reported that improved overall survival outcomes in patients with CRC were correlated with elevated gene expression of H2R and decreased gene expression of H1R in the intestinal mucosa (Shi et al., 2019). The study further demonstrated that histamine-producing *L. reuteri* can suppress tumorigenesis in an established CRC mouse model but compared to H1R antagonists, H2R antagonists dramatically increased both the size and number of tumors (Shi et al., 2019). Given these data, different receptors of histamine may play distinct roles in the gut.

#### 3.2.3 Exopolysaccharide

Exopolysaccharide (EPS), a substance generated by *L. reuteri*, is essential for the development of biofilms and *L. reuteri*’s adhesion to surfaces of the epithelium (Wang et al., 2021b). And *in vitro E. coli* adherence to porcine epithelial cells was also inhibited by EPS synthesized by *L. reuteri* (Ksonzekova et al., 2016). Consequently, the gene expression of proinflammatory cytokines like TNF-α and IL-6 brought on by *E. coli* or *Salmonella Typhimurium* infection was suppressed by EPS derived from *L. reuteri* (Ksonzekova et al., 2016; Kissova et al., 2022).

#### 3.2.4 Short-chain fatty acids

SCFAs are a set of metabolite products derived from *L. reuteri* and engaged in regulating immune response and inflammatory reactions, thereby, promoting intestinal health (Smith et al., 2013; Oh et al., 2021). Intestinal SCFAs mainly consist of acetate, propionate, and butyrate, which can be generated by *L. reuteri* in different situations (Liu et al., 2022c; Calvigioni et al., 2023; Van den Abbeele et al., 2023). SCFAs are also suggested to be essential for ameliorating gut microbiota dysbiosis in mice colitis treated with *L. reuteri* (Lee et al., 2022). Particularly, butyrate is the typical metabolite which can enhance gastrointestinal motility by mediating the enteric neurons and maintaining the proliferation of Cajal cells though the AKT-NF-κB pathway (He et al., 2020). Besides, *L. reuteri* was reported to exert anti-tumor effects in mice with hepatocellular carcinoma by upregulating acetate levels (Hu et al., 2023). Recently propionate and butyrate were also reported to suppress the adhesion and invasion ability of *Salmonella Typhimurium in vitro* experiments (Liu et al., 2022b).

Together, these observations provide substantial evidence that metabolites generated by *L. reuteri* are involved in the regulation of gut immune response and remodeling of the commensal microbiota composition via the activation of various signal pathways.

### 3.3 Enhancement of intestinal epithelial barrier

Numerous studies showed that intestinal epithelial barrier damage disrupts immune homeostasis and contributes to many intestinal disorders, especially IBD and CRC (Leibovitzh et al., 2023). The capacity of *L. reuteri* to boost gut barrier function has been demonstrated in several investigations (Yi et al., 2018; Gao et al., 2022). On one hand, *L. reuteri* owns the ability to maintain the intestinal epithelial repair function. Wu and colleagues found that *L. reuteri* reconstructed the epithelial integrity by activating the Wnt/β-catenin pathway, thereby promoting the intestine epithelial cell proliferation and inducing intestinal stem cell differentiation to Paneth cells, even under the condition of TNF-induced intestinal inflammation (Wu et al., 2020). On the other hand, *L. reuteri* was also reported to maintain the integrity of the gut barrier by upregulating the expression of TJ proteins, evidenced in in preclinical studies (Gao et al., 2022; Liu et al., 2022a; Li et al., 2023a). TJ proteins are primary for epithelial cell proliferation and gut permeability (Kuo et al., 2021). Bacterial extracellular membrane vesicles derived from *L. reuteri* contain lipoteichoic acid, identified as a Toll-like receptor (TLR) 2 activator (Pang et al., 2022). According to research, TLR2 activation strengthened the intestinal barrier by increased expression of TJ proteins via the PI3K-Akt-signaling pathway (Gu et al., 2016). *L. reuteri* was also reported to induce TJ proteins expression via the activated PKC-Nrf-2/HO-1pathway and inhibited NF-κB pathway, along with reduced apoptosis of intestinal epithelial cells (Zhou et al., 2022b). In conclusion, these results indicated the therapeutic benefits of *L. reuteri* in gastrointestinal diseases by repairing the gut barrier function though different pathways.

### 3.4 Regulation of immune cell differentiation and function

*L. reuteri* may exhibit beneficial effects in digestive system diseases via modulation of the immune system. DCs are crucial in the interaction between microorganisms and gut immune responses because of their advantageous location and capacity to present luminal antigens as “gatekeepers” (Saez et al., 2023). Mature DCs promote the polarization of naive T cells toward T helper (Th) 1, Th2, Th17, or Treg responses (Tiberio et al., 2018). *L. reuteri* 5454 treatment alleviated colitis inflammation in mice, the potential molecular mechanism might be explained by the efficiency of DCs primed with *L. reuteri* 5454 to promote Tregs differentiation and trigger IL-22 secretion (Hrdy et al., 2020). Another study revealed that *L. reuteri* 17938 may activate DCs via TLR2 and lead to Tregs expansion in the intestinal mucosa, thereby alleviating experimental necrotizing enterocolitis (Hoang et al., 2018). Further evidenced by *in vitro* experiments, *L. reuteri* cell-free supernatants promoted DCs differentiation with up-regulated surface markers (CCR7, CD83, CD86, HLA-DR) and enhanced cytokine synthesis (IL-6, IL-10, and IL-23), nonetheless, these DCs exhibited diminished phagocytic activity (Haileselassie et al., 2016). The similar results were also reported by Lasaviciute and his colleagues who showed that a mixed secondary response profile was produced in DCs after priming human monocytes with *L. reuteri* DSM17938 secretions, secreting low levels of TNF-α, and IL-27 and high levels of IL-1β and IL-6 (Lasaviciute et al., 2022). The particular manner by which *L. reuteri* modulates the phenotypic and operation of mucosal-like DC is yet fully understood. Notably, the involvement of surface proteins was examined recently in the interaction between *L. reuteri* and DCs. The study demonstrated that *L. reuteri* can promote the release of the immune-regulating IL-10 in monocyte-derived DCs independent of its mucus adhesins while the production of pro-inflammatory cytokines (IL-6, TNF-α, IL-1β) was enhanced by mucus adhesins (Bene et al., 2017).

Meanwhile, numerous preclinical studies showed *L. reuteri* also had the property to promote the proliferation of Tregs while suppressing the production of Th cells, specifically Th1 and Th2, accompanied by decreased mRNA expression of NF-κB, TLR4, TNF-α, IL-6, and upregulated IL-10 mRNA expression in the intestine (Hoang et al., 2018; Liu et al., 2019; Liu et al., 2023b). The forkhead box P3 (FoxP3) transcription factor is crucial in generating Tregs (Harada et al., 2022). And the FoxP3 mRNA expression level was shown to be elevated in peripheral blood after *L. reuteri* DSM 17938 administration in colicky infants (Savino et al., 2018b). In addition, the mechanism of Tregs induction may be associated with reprogrammed gut microbiota composition and SCFAs production by *L. reuteri* (Liu et al., 2019; Wen et al., 2021). However, the ability of *L. reuteri* to induce Tregs is generally strain-dependent, and certain *L. reuteri* strains have anti-inflammatory properties that are not always reliant on Tregs (Liu et al., 2021b; Liu et al., 2023a). Orally feeding *L. reuteri* can suppress Th1/Th2 responses in mesenteric lymph nodes in Treg-deficient mice via activation of adenosine A_2A_ receptors (Liu et al., 2023a). This phenomenon may be explained by the specific property of *L. reuteri* to increase the level of plasma adenosine metabolites such as inosine (Liu et al., 2021b). Proteomics studies using mass spectrometry confirmed that bacterial extracellular membrane vesicles derived from *L. reuteri* carried numerous bacterial cell surface proteins, including 5’-nucleotidase, which initiate the process by which AMP is transformed into the signal molecule adenosine (Pang et al., 2022).

Besides, *L. reuteri* seemed to be able to promote M2 macrophage polarization. Pro-inflammatory M1-like macrophage indicators may be suppressed by the stress protein GroEL isolated from *L. reuteri*, whereas M2-like markers are favored, consequently suppressing intestinal production of pro-inflammatory markers (TNF-α, IL-1β, interferon-γ) and increasing anti-inflammatory IL-10 and IL-13 by the activation of a TLR4 pathway (Dias et al., 2021). Except for these, Wang and colleagues reported that oral therapy of *L. reuteri* effectively inhibited the progression of mice colitis induced by immune checkpoint blockade treatment, and they highlighted that the therapeutic impact of *L. reuteri* ATCC PTA 6475 was related to a decrease of ILC3s (Wang et al., 2019). ILC3s are mainly found in the intestinal mucosa and can drive proinflammatory responses and cause immunopathological damage (Jarade et al., 2021). The mechanism is not fully clear. It is possible that *L. reuteri*’s capacity to metabolize tryptophan into indole derivatives, which subsequently activate the aryl hydrocarbon receptor in ILC3s, is responsible for the underlying mechanism (Zelante et al., 2013; Schiering et al., 2017). Furthermore, a previous study investigated two strains extracted from piglets, *L. reuteri* ZJ617 and *L. reuteri* ZJ615, characterized by high and low adhesive ability respectively (Gao et al., 2016). The study showed that *L. reuteri* ZJ615 reduced phosphorylation levels of ERK1/2 while *L. reuteri* ZJ617 had no significant effects on the ERK1/2 signaling pathway in the ilea of the LPS-stimulated mice model (Gao et al., 2016). Given this, *L. reuteri*’s capacity to modulate the immune system may be somewhat dependent on its adhesive property.

Overall, these studies demonstrated that metabolites and bacterial components of *L. reuteri* as well as the restored balance of the gut microbiota are both engaged in the suppression of excessive immune response and protection the intestine from injury.

## 4 Conclusions and future perspectives

In the past decades, research regarding the link between digestive diseases and the gut microbiome is growing quickly, accompanied by increased interest in probiotics in gut health. *L. reuteri* may serve as a viable candidate for the treatment of digestive system disorders owing to its potent antimicrobial, immunomodulatory, and anti-inflammatory activities with nearly no safety risks.

In this review, we systematically interpreted its application in different digestive disorders, including IC, diarrhea and constipation, FAP, *H. pylori* infection, IBD, and liver diseases. The results discussed here showed that *L. reuteri* may alleviate the symptoms of digestive problems through a variety of mechanisms, such as manipulation of the intestinal microbial population, barrier function of the epithelium, regulation of immunity, and modulation of numerous metabolites. Most of the *in vitro* and *in vivo* experiments revealed beneficial impacts of *L. reuteri* but the results of some clinical trials may be controversial. This phenomenon may be explained, in part, by specific and different functions of multiple stains and the high diversity of the human gut microbiota affected by sex, population, diet as well as other factors.

According to well-established studies, *L. reuteri* treatment is effective in improving IC, diarrhea, constipation, and *H. pylori* infection, whether to be served as a monotherapy or adjuvant strategy. While given the limited clinical data, there is no sufficient support data to recommend *L. reuteri* application in FAP, IBD, diverticulitis, CRC and liver diseases. In conclusion, it is important to conduct more research on *L. reuteri*’s potential benefits for treating digestive system diseases so that it may be used in clinical settings and as a therapeutic strategy.

## Author contributions

RY: Conceptualization, Funding acquisition, Methodology, Project administration, Resources, Supervision, Visualization, Writing – review & editing. YP: Writing – original draft. YM: Funding acquisition, Writing – original draft. ZL: Conceptualization, Writing – review & editing. YJ: Software, Writing – review & editing. ZX: Methodology, Supervision, Validation, Writing – review & editing.
